# Supplementary figures and images for: Astrocytes derived from neural progenitor cells are susceptible to Zika virus infection
Source: PLoS One. 2023 Mar 29;18(3):e0283429. doi: 10.1371/journal.pone.0283429 (PMC10057746; doi:10.1371/journal.pone.0283429)

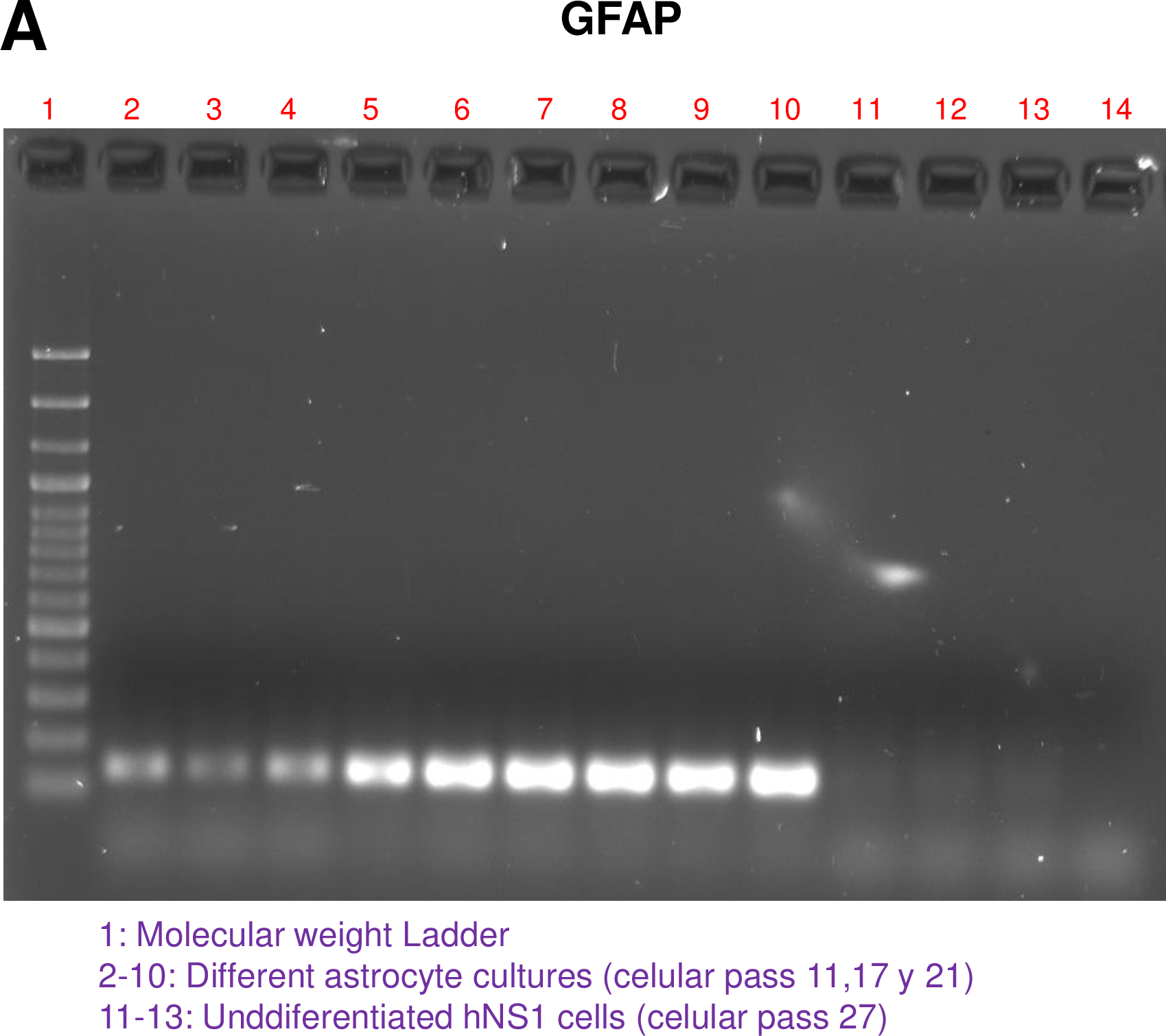

Supplement: S1 Fig — The astrocytes-hNS1 culture was seeded. At three days of proliferation, cellular RNA was recovered by analyzing the expression of gene markers of glial cells. Expression of GFAP in cells astrocytes-hNS1 and hNS1 cell line. (TIF) [file pone.0283429.s001.tif]
