# Supplementary material for: Astrocytes derived from neural progenitor cells are susceptible to Zika virus infection
Source: PLoS One. 2023 Mar 29;18(3):e0283429. doi: 10.1371/journal.pone.0283429 (PMC10057746; doi:10.1371/journal.pone.0283429)
Supplement: S1 Table — Primers used and their characteristics. (DOCX) [file pone.0283429.s002.docx]

| GENE | ENCODED | PRIMERS SEQUENCE (5’-3´) | | AMPLICON |
| --- | --- | --- | --- | --- |
| *APOE* | APOE | FORWARD | GGAAGGCTAACCTGGGACTG | 198 |
|  |  | REVERSE | ATCCCAAAAGCGACCCAGTG |  |
| *AXL* | AXL | FORWARD | GCTTCGGGATGGACAGATCC | 216 |
|  |  | REVERSE | AAGTAAGGCAAGCCCTCCAG |  |
| *GJA1* | CX43 | FORWARD | CAAAATCGAATGGGGCAGGC | 136 |
|  |  | REVERSE | GCTGGTCCACAATGGCTAGT |  |
| *SLC1A3* | EAAT1 | FORWARD | CGCCATCTTTATAGCCCAAA | 139 |
|  |  | REVERSE | CAGAATGAGGAGCATGGTGA |  |
| *SLC1A2* | EAAT2 | FORWARD | CATGCACAGAGAAGGCAAAA | 210 |
|  |  | REVERSE | AGAGTCTCCATGGCCTCAGA |  |
| *GAPDH* | GAPDH | FORWARD | CAAGGCTGAGAACGGGAAGC | 194 |
|  |  | REVERSE | AGGGGGCAGAGATGATGACC |  |
| *GFAP* | GFAP | FORWARD | ACATCGTGGTGAAGACCGTG | 142 |
|  |  | REVERSE | CTATCCTGCTTCTGCTCGGG |  |
| *GLUL* | GS | FORWARD | GCTGGTGTAGCCAATCGTAGC | 123 |
|  |  | REVERSE | GGCTTCTGTCACCGAAAAGG |  |
| *MERTK* | MERTK | FORWARD | AAATCCCCCTCCGTGCTAAC | 129 |
|  |  | REVERSE | TGGGGAGGGAATTGCTTTGAT |  |
| *GRIN1* | NMDAR | FORWARD | AGCTTCTACAACACCGAGGC | 203 |
|  |  | REVERSE | GAGGCGTACACGATCTCCAG |  |
| *PPAR-γ* | PPAR-γ | FORWARD | GCCTTAACCTCTGCTGGTGA | 140 |
|  |  | REVERSE | TCGTTAAAGGCTGACTCTCGTT |  |
| *TYRO-3* | TYRO-3 | FORWARD | GACTGGTCCTGAGAGGGTGA | 226 |
|  |  | REVERSE | CCCATGAGCTTCAGACCCTTT |  |
| *ZIKV (non-structural protein 1, NS1)* | ZIKV (non-structural protein 1, NS1) | FORWARD | GAGTGTGATCCAGCCGTTATT | 105 |
|  |  | REVERSE | CAGCCTCCATGTGTCATTCT |  |

**S1 Table. The Primers used for PCR.** Primers used and their characteristics.
